# Vacuum-Assisted Closure Treats Refractory Esophageal Leak in a Pediatric Patient

**DOI:** 10.7759/cureus.35257

**Published:** 2023-02-21

**Authors:** Evan K Lin, Felicia Lee, Jasmin Cao, Christian Saliba, Vivian Lu, Raymond I Okeke, Justin Sobrino, Christopher Blewett

**Affiliations:** 1 Medical School, Saint Louis University School of Medicine, Saint Louis, USA; 2 Pediatric Surgery, SSM Health Cardinal Glennon Children's Hospital, Saint Louis, USA; 3 General Surgery, SSM Health Saint Louis University Hospital, Saint Louis, USA

**Keywords:** wound vac, esophageal leak, endoluminal vacuum, esophagus perforation, esophagus

## Abstract

Esophageal perforations can have iatrogenic and non-iatrogenic causes. Early identification is a predictor of good outcomes. When identified, perforations can be managed conservatively with wide drainage or repaired surgically. Endoscopic esophageal vacuum-assisted closure may be used as a definitive treatment, particularly in scenarios where conservative management and primary surgical repair fail to achieve complete healing. We present such a scenario advocating for the consideration of endoscopic esophageal vacuum-assisted closure in patients with refractory esophageal leaks.

## Introduction

Esophageal perforations, although rare, can occur following foreign body ingestion, intubation, or endoscopic instrumentation in pediatric patients. Complications can be fatal and are associated with significantly increased mortality rates of up to 20-30% [1]. Around 71-84% of esophageal perforations (EP) occur due to iatrogenic causes in the pediatric population. Nonoperative management involves maximal nutritional support, controlling local contamination with broad-spectrum antibiotics, and maintaining good esophageal patency. Surgical management, including debridement and drainage with direct repair by suturing, is reserved for patients with persistent clinical deterioration or massive leaks or when identified in the first 24 hours [1,2]. Minimally invasive approaches may be used to treat EP. Such approaches include endoscopic stenting, clipping, and, most recently, endoscopic esophageal vacuum-assisted closure (EVAC) [2-4].

## Case presentation

**Figure 1:**
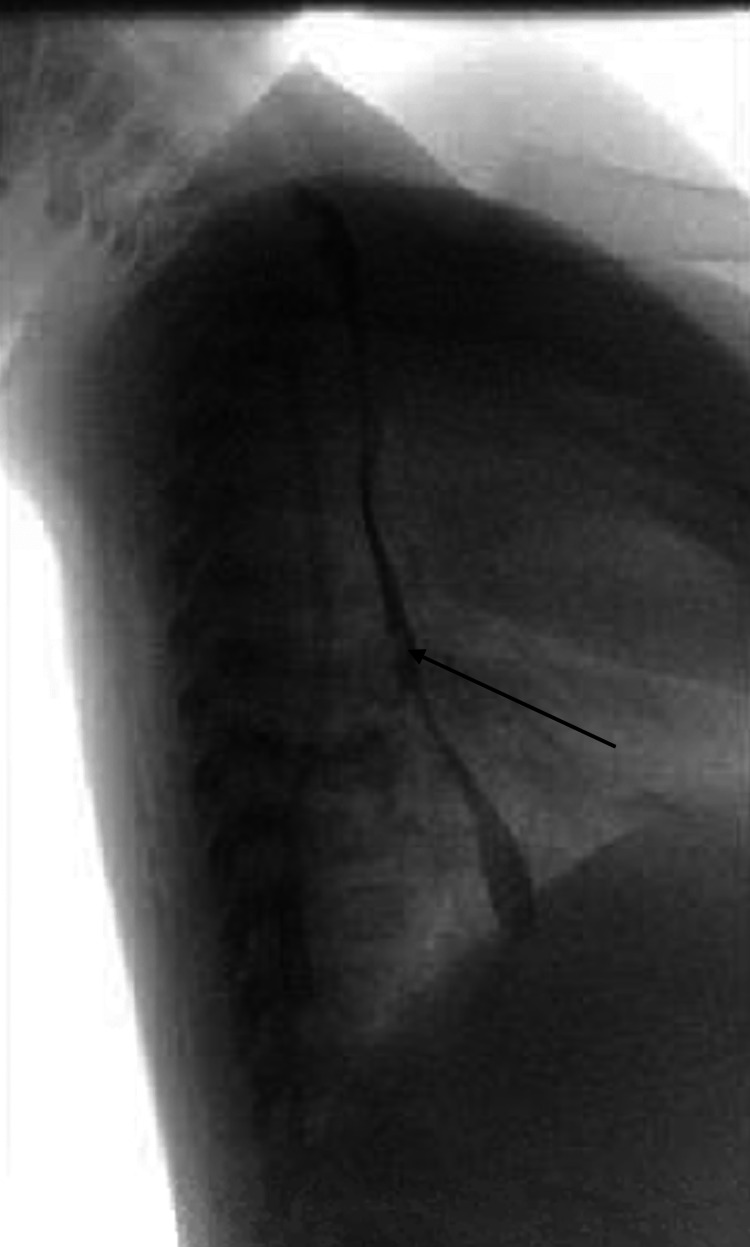
Esophagram showing mid-esophagus leak (black arrow).

**Figure 2:**
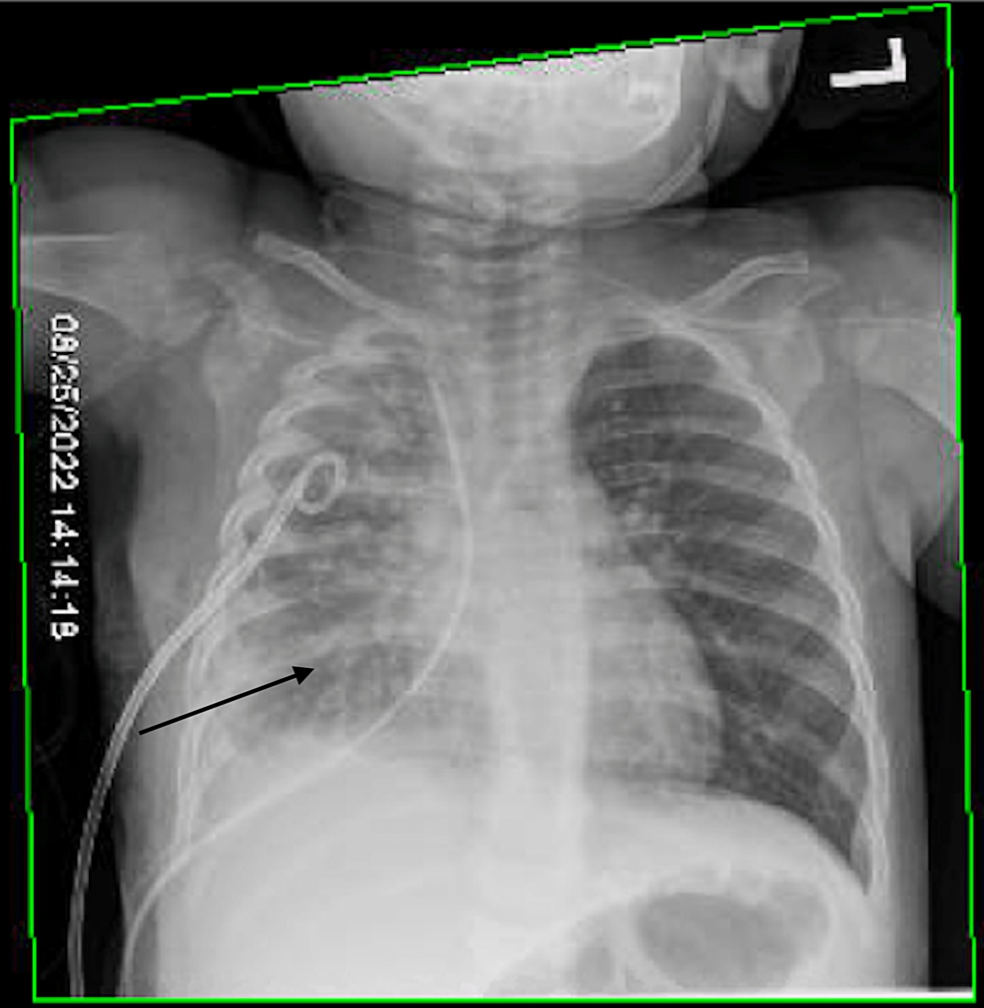
Chest X-ray showing right-sided pleural effusion (black arrow).

**Figure 3:**
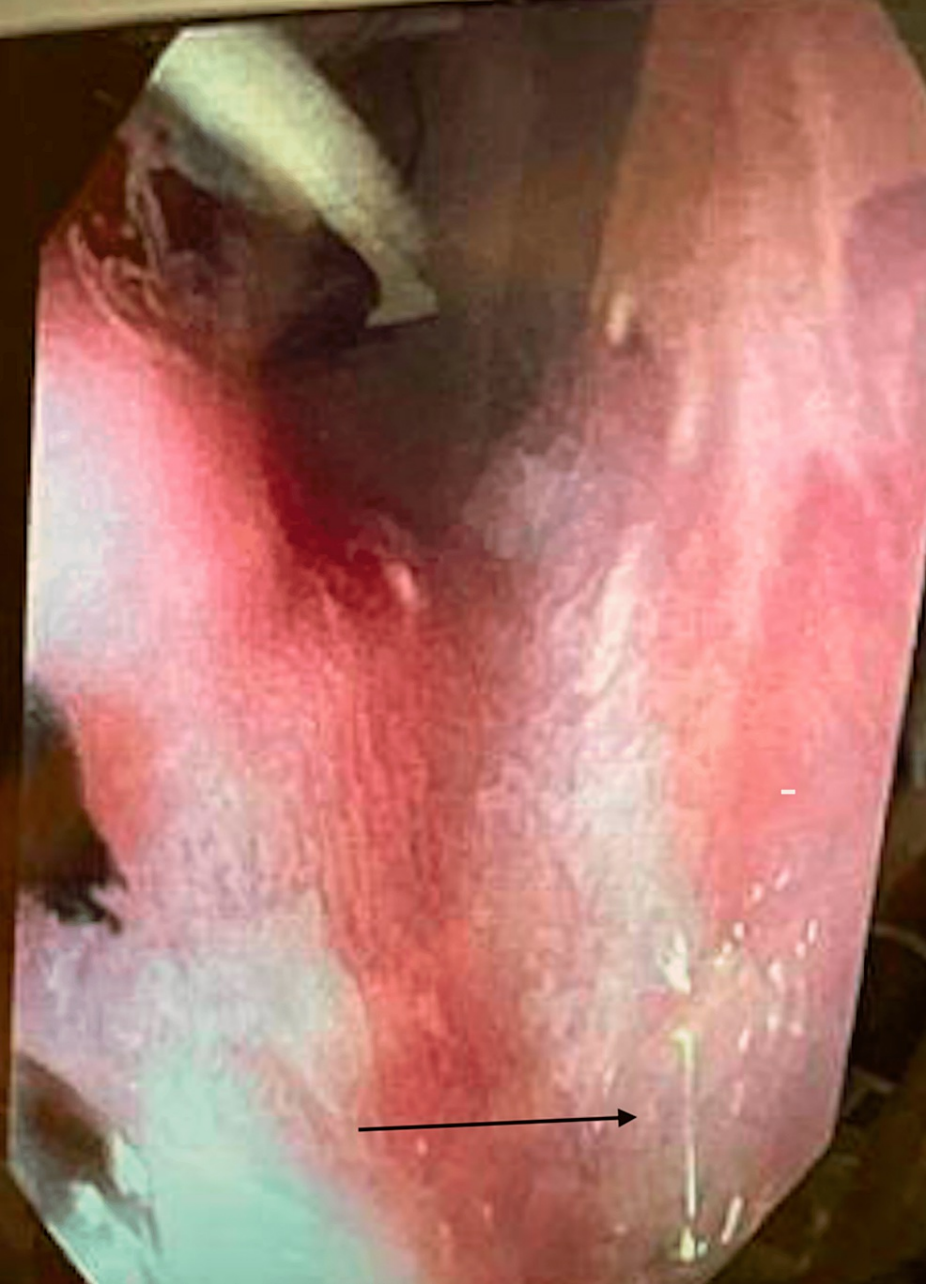
Esophagogastroduodenoscopy showing esophageal perforation (black arrow).

**Figure 4:**
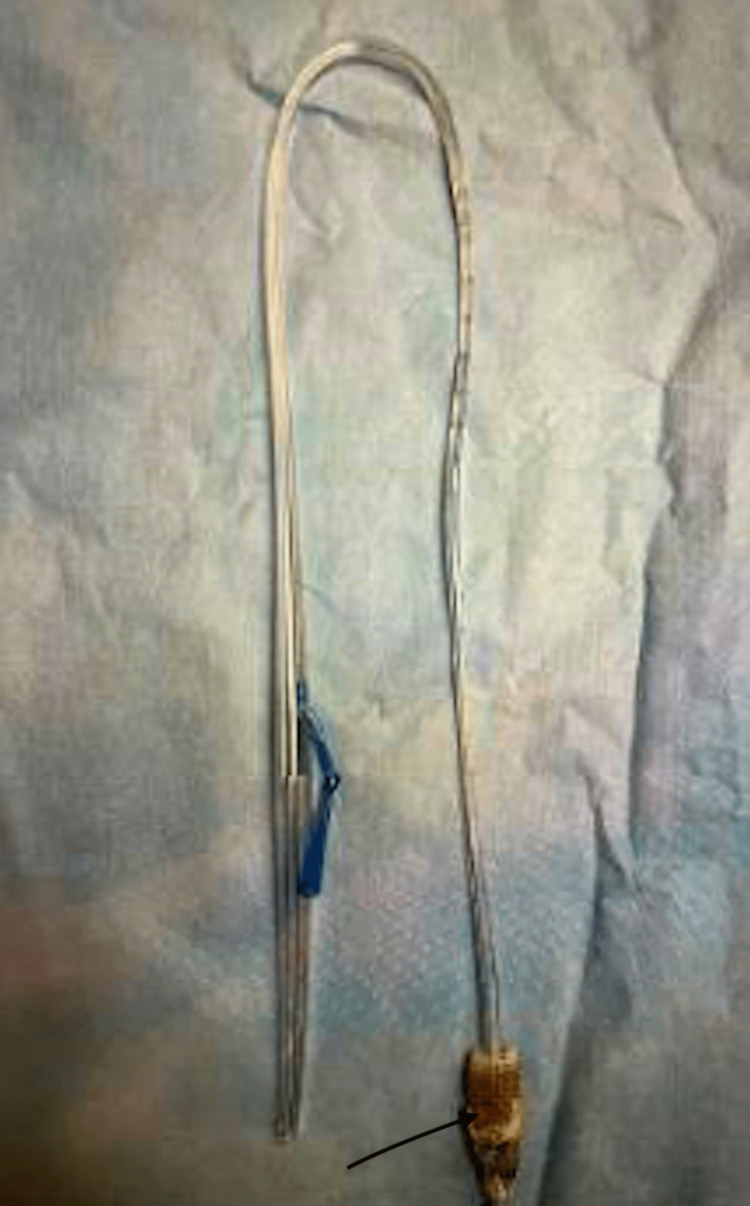
An endoluminal esophageal vacuum (EndoVac) device. The sponge is highlighted by the black arrow.

**Figure 5:**
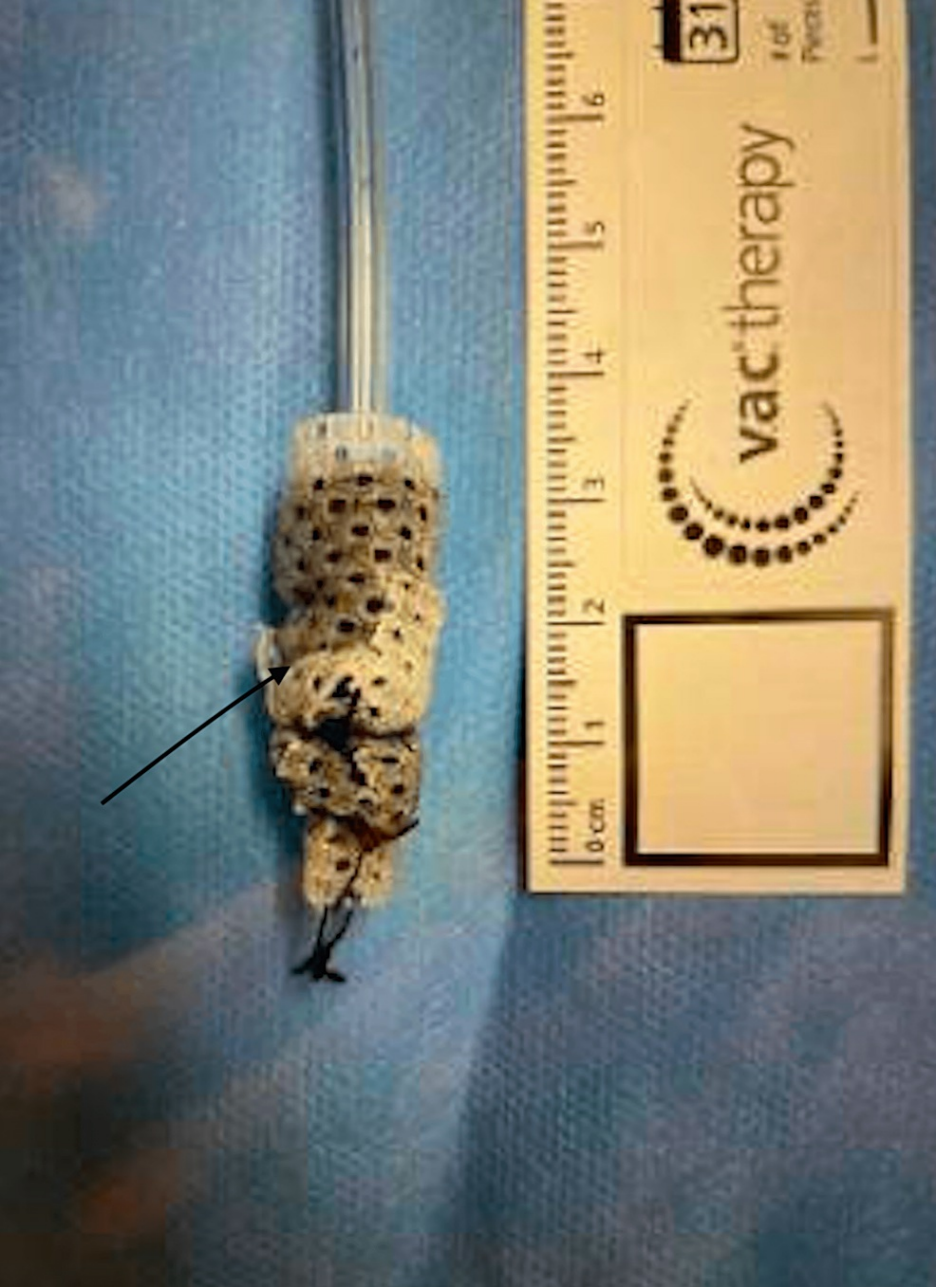
Black sponge wrapped with a nonadhesive gauze and then sutured to a 10 French nasogastric (NG) tube.

A six-month-old patient presented at our institute for scheduled surgical repair of a congenital esophageal duplication cyst diagnosed on computed tomography (CT). After an uncomplicated thoracoscopic resection, a planned esophagram showed a leak of contrast at the resection site on a post-operative day (POD) 1. The patient returned to the operating room (OR) that same day for a thoracoscopic primary repair. A full-thickness defect was identified in the prior cyst repair resection bed, which we primarily repaired. On POD 3 after this repair, the patient developed tachypnea. A chest X-ray (CXR) showed a large anterior pneumothorax and possible right-side pleural effusion. The patient was taken back to the OR for chest tube placement. On POD 9, a routine esophagram showed a persistent mid-esophageal leak (Figure 1). After two weeks of conservative management with total parenteral nutrition (TPN) and staying nil per os (NPO) with a nasogastric (NG) tube in place, a repeat esophagram showed persistence of the esophageal leak and a CXR showed a right-sided pleural effusion (Figure 2). The leak persisted on repeat study a week later. We planned to place an endoscopic esophageal vacuum device (EVAC) and a nasoduodenal (ND) feeding tube. During the procedure, the esophagogastroduodenoscopy (EGD) showed a sub-centimeter cone shape defect (Figure 3), with evidence of fluid traversing the lesion. To make the EVAC possible, we sutured a black wound vac sponge around a 10 French nasogastric (NG) tube and wrapped around it a nonadhesive gauze to protect the esophageal mucosa from the sponge (Figures 4-5). The EVAC was inserted from the patient’s mouth and applied near the perforation site. The patient tolerated the procedure well without any complications. EVAC was replaced twice over the next seven days. It was displaced on Day 8 from an episode of uncomplicated emesis following increased feeds. The following esophagram showed no contrast extravasation, and we started the patient on an oral diet. The patient’s right pleural effusion had also decreased in size. We then pulled the chest tube, and the patient tolerated oral intake. We discharged the patient in stable condition after 35 days of hospitalization.

## Discussion

In 2011, Loske et al. and Schorsch et al. introduced EVAC to manage EP in the adult population [2,5]. This treatment method adds to the algorithm proposed by Kaman et al. for EP resolution [6]. Multiple studies have since confirmed its success at closure, with rates ranging from 70-100% [7]. EVAC relies on applying negative pressure for healing. Negative pressure facilitates closer apposition and closure of the wound edges by granulation tissue. It creates changes in regional blood flow stimulating local angiogenesis and subsequently increases oxygen delivery to the wound. Furthermore, it minimizes the risk of infection by actively draining gastroesophageal contents and preventing fluid secretions from leaking into the chest cavity [7,8].

While EVAC has become the standard of care in many adult institutions, adoption in pediatrics has lagged behind. Manfredi et al. in 2018 showed the successful application of EVAC in pediatrics [7]. Patients with EP either underwent EVAC or esophageal stent therapy. EVAC was comparable to stenting in cases of iatrogenic EP and EVAC is superior to stenting in surgical anastomotic perforations [7,8]. Furthermore, stenting has complications like stent migration, malpositioning, and obstruction [1,9]. In this case study, our patient developed iatrogenic EP after the surgical resection of an esophageal duplication cyst with subsequent development of right pleural effusion. We repaired the EP and inserted a chest tube to facilitate drainage. However, due to persistent esophageal leaks after surgical and conservative management, EVAC was eventually inserted with a successful resolution. A 10 Fr oral-esophageal tube attached to a sponge on the tip and wrapped with Safetac was placed at the perforation site as endoluminal VAC. The device was not factory designed.

Kaczmarek et al. [10] specifically looked at infants at 7-159 days of life and found that in all four of their cases, EVAC led to complete EP closure after 22 days and an average of 4.5 EVAC exchanges. Fraga et al. looked at an eight-month-old male with esophageal perforation after a video-assisted thoracoscopic lobectomy for a right lower lobe congenital malformation [11]. They first pursued surgical repair, followed by EVAC due to persistent esophageal leakage with a successful resolution. Ritz et al. [12] suggested that while EVAC significantly reduced inflammation and the size of the perforation, complete closure of EP cannot be accomplished by EVAC alone, and their patients (median age 3.5-years-old) required subsequent ArgyleTM Replogle Suction Catheters (Cardinal Health, Inc., Dublin, OH) or surgical repair.

Our case is an addition to the successful examples in the literature of EVAC application for pediatric patients. These cases altogether contribute to the literature suggesting the feasibility of EVAC in very young pediatric patients and its success in repairing EP. However, these cases also demonstrate the need to compare the success rate and secondary outcomes of surgical repair vs. EVAC as first-line therapy. There is great promise in EVAC therapy for young patients, including infants, for EP resolution.

## Conclusions

Esophageal perforations can lead to sepsis, multi-organ failure, and death. In recent years, minimally invasive techniques like EVAC and esophageal stenting have emerged as first-line therapy for EP in the adult population. Since the successful application of EVAC in pediatrics in 2018, several studies have shown the feasibility and success of esophageal closure with EVAC. Our case study adds to this growing literature, demonstrating that we can safely consider EVAC a treatment option in select pediatric patients.
